# Herd-level seroprevalence, molecular prevalence, and trends of *Coxiella burnetii* (Q fever) in cattle worldwide: A systematic review and meta-analysis

**DOI:** 10.14202/vetworld.2024.2811-2828

**Published:** 2024-12-18

**Authors:** Adithep Konputtar, Nguyen Hoai Nam, Sarinya Rerkyusuke, Chaiyapas Thamrongyoswittayakul, Suvaluk Seesupa, Montira Yossapol, Peerapol Sukon

**Affiliations:** 1 Veterinary Science Program, Faculty of Veterinary Medicine, Khon Kaen University, Khon Kaen, 40002, Thailand; 2 Department of Animal Surgery and Theriogenology, Faculty of Veterinary Medicine, Vietnam National University of Agriculture, Trauqui, Gialam, Hanoi, Vietnam; 3 Division of Livestock Medicine, Faculty of Veterinary Medicine, Khon Kaen University, Khon Kaen, 40002, Thailand; 4 KKU Research Program, Khon Kaen University, Khon Kaen, 40002, Thailand; 5 Division of Theriogenology, Faculty of Veterinary Medicine, Khon Kaen University, Khon Kaen, 40002, Thailand; 6 Veterinary Infectious Disease Research Unit , Faculty of Veterinary Sciences, Mahasarakham University, Maha Sarakham, 44150, Thailand; 7 Division of Anatomy, Faculty of Veterinary Medicine, Khon Kaen University, Khon Kaen, 40002, Thailand

**Keywords:** cattle, *Coxiella burnetii*, herd, prevalence, Q fever

## Abstract

**Background and Aim:**

Cattle are the reservoir host of *Coxiella burnetii*, a causative agent of Q fever. Pooling herd-level prevalence data from individual studies would help determine the global prevalence of *C. burnetii* in cattle herds. This study aimed to estimate the global herd-level seroprevalence and molecular prevalence of *C. burnetii* in cattle, explore sources of heterogeneity, and determine trends and cumulative evidence of the pooled prevalence over time.

**Materials and Methods:**

Relevant studies were retrieved from PubMed, Scopus, and Web of Science and then screened for possible inclusion. A random-effects model was used for all meta-analyses. Subgroup meta-analysis and meta-regression were used to explore some sources of heterogeneity associated with the pooled prevalence and to determine the trends of *C. burnetii* in cattle herds over the study years (1961–2020). A cumulative meta-analysis was used to determine the cumulative evidence of the pooled prevalence over the publication years.

**Results:**

Of the 1541 citations, 86 studies with 38,057 cattle herds from 42 countries on six continents were included in the meta-analysis. The global herd-level seroprevalence of *C. burnetii* in cattle was estimated to be 44.4% (95% confidence interval [CI], 37.9%–51.1%), with high heterogeneity among the included studies. The herd-level seroprevalence was significantly higher in dairy than in beef cattle herds (49.0% [95% CI: 41.9%–56.2%] vs. 14.5% [95% CI: 5.8%–32.1%], respectively). The global herd-level molecular prevalence of *C. burnetii* in cattle was estimated to be 32.3% (95% CI: 25.3%–40.01%), with high heterogeneity among the included studies. Herd-level molecular prevalence was significantly different among continents. The herd-level molecular prevalence ranged from 12.8% (95% CI: 7.1%–21.9%) in Asia to 70.0% (95% CI: 36.3%–90.5%) in North America. Regarding trends, the herd-level seroprevalence of *C. burnetii* in cattle did not change significantlyover the study years.

**Conclusion:**

The global herd-level seroprevalence and herd-level molecular prevalence of *C. burnetii* in cattle were high, estimated at 44% and 32%, respectively. The herd-level seroprevalence trend did not significantly change over time. This result indicates that cattle remain a major reservoir host for *C. burnetii* and pose a potential risk to human health.

## Introduction

Q fever is a worldwide zoonotic disease caused by *Coxiella burnetii*, a Gram-negative and obligate intracellular bacterium belonging to the family *Coxiellaceae*, class *Gammaproteobacteria*, and phylum *Proteobacteria* [1]. *C. burnetii* is a highly contagious agent due to its high durability, high infectivity, and easy transmission. Thus, *C. burnetii* is an emerging pathogen and a potential bioterrorism agent [2]. In humans, infected patients may experience acute, self-limiting, and nonspecific symptoms, including elevated body temperature, intense headache, exhaustion, vomiting, and pneumonia; alternatively, these patients may develop chronic symptoms, such as endocarditis, hepatitis, osteomyelitis, encephalitis, and abortion in pregnant women [3, 4]. In addition, co-infection of *C. burnetii* with other diseases, such as malaria, tuberculosis, and coronavirus disease 2019, has also been observed [3, 5, 6]. This co-infection may lead to complications in the treatment and care of infected patients. In animals, *C. burnetii* infections (also known as coxiellosis) occur in several animal hosts, such as mammals, birds, reptiles, and arthropods [1, 7]. Both domestic and wild animals can be infected with *C. burnetii*. In domestic animals, several species, such as ruminants, rabbits, dogs, cats, horses, pigs, and camels, have been reported to have *C. burnetii* infection [8]. Over 100 wildlife mammal species can be infected and act as reservoir hosts for *C. burnetii* [9].

According to zoonotic evidence, small ruminants are recognized as the primary source of *C. burnetii* infections in humans because many Q fever outbreaks in humans have been linked to small ruminants [10–12]. However, several small Q fever outbreaks in humans have been reported, linked to cattle [13–15]. Infected ruminants (especially sheep, goats, and cattle) can excrete *C. burnetii* through several routes, such as through milk, amniotic fluid, urine, vaginal mucus, and feces [16]. Research findings suggest that *C. burnetii* is commonly shed by vaginal discharge in sheep, whereas milk is the predominant route of pathogen shedding in cattle and goats [11, 17]. Most infected ruminants are asymptomatic, but *C. burnetii* can cause late-term abortion, stillbirth, and weak offspring in sheep and goats and can cause mastitis, metritis, premature birth, and infertility in cattle [11, 16, 18].

Cattle are among the most important livestock species raised for meat and milk worldwide. The global cattle population increased from 942 million in 1961 to more than 1500 million in 2020 [19]. Most cattle infected with *C. burnetii* are asymptomatic, and these cattle can shed the pathogen to the environment (especially through milk), which may result in animal-to-animal transmission within a herd and create zoonotic risks for humans [14, 15, 20]. Therefore, herd health management, such as surveillance and monitoring systems using serological or polymerase chain reaction (PCR) tests to detect evidence of *C. burnetii* infection in herds, is crucial for eliminating and controlling the disease [11]. Previous studies [21–27] have reported the herd-level seroprevalence and herd-level molecular prevalence of *C. burnetii* infections in cattle worldwide. However, individual studies have some limitations in terms of the study locations, study years, and the number of sample sizes. Combining the results from the relevant individual studies would help to draw a global picture of the prevalence of *C. burnetii* in cattle herds.

Therefore, this study aimed to estimate the global herd-level seroprevalence and molecular prevalence of *C. burnetii* in cattle worldwide, explore some sources of heterogeneity associated with these prevalences, and determine the trends and cumulative evidence of the herd-level prevalence of *C. burnetii* in cattle herds over time.

## Materials and Methods

### Ethical approval

Due to the absence of animal involvement or the use of animal-derived products, ethical approval was not required. This study was conducted in accordance with the principles of the Preferred Reporting Items for Systematic Reviews and Meta-Analyses [28]. The protocol was prepared before study commencement and can be obtained from the corresponding author upon request.

### Study period and location

The literature search, data collection, and data analysis were conducted at the Faculty of Veterinary Medicine, Khon Kaen University, from January 2022 to August 2023. The included studies were published between 1964 and 2023. The included studies were conducted in 42 countries from Africa, Asia, Australia, Europe, North America and South America.

### Search strategies

Two authors searched three citation databases (PubMed, Scopus, and Web of Science) to identify scientific papers documenting the prevalence of Q fever in cattle. The search covered the entire database up to August 15, 2023. The search involved the use of specific keywords linked to *C. burnetii*, Q fever, prevalence, and cattle. The search was limited to articles written in English. Medical subject headings (MeSH) were verified to ensure the use of appropriate keywords. The search details from the databases are as follow: (“*Coxiella burnetii*”[All Fields] OR “Q fever”[All Fields]) AND (“epidemiologies”[All Fields] OR “epidemiology”[MeSH Subheading] OR “epidemiology”[All Fields] OR “epidemiology”[MeSH Terms] OR “epidemiology s”[All Fields] OR (“epidemiology”[MeSH Subheading] OR “epidemiology”[All Fields] OR “surveillance”[All Fields] OR “epidemiology”[MeSH Terms] OR “surveillance”[All Fields] OR “surveillances”[All Fields] OR “surveilled”[All Fields] OR “surveillance”[All Fields]) OR “Sero-prevalence”[All Fields] OR (“epidemiology”[MeSH Subheading] OR “epidemiology”[All Fields] OR “prevalence”[All Fields] OR “prevalence”[MeSH Terms] OR “prevalence”[All Fields] OR “prevalences”[All Fields] OR “prevalence s”[All Fields] OR “prevalent”[All Fields] OR “prevalently”[All Fields] OR “prevalents”[All Fields])) AND (“cattle”[MeSH Terms] OR “cattle”[All Fields] OR (“dairies”[All Fields] OR “dairy”[All Fields] OR “dairy s”[All Fields] OR “dairying”[MeSH Terms] OR “dairying”[All Fields]) OR (“cattle”[MeSH Terms] OR “cattle”[All Fields] OR “cow”[All Fields])) The relevant entries from each database were merged, and redundant entries were discarded.

### Study selection

The inclusion criteria were studies that provided data on the prevalence of *C. burnetii* or Q fever in cattle at the herd level, specifically from cross-sectional or survey studies. Articles or studies were excluded if they fell into the following categories: (1) Reviews, (2) case reports, (3) experimental studies, (4) articles lacking clear data on prevalence, and (5) articles reporting Q fever at the individual cattle level. The process of selecting studies involved two distinct stages. The first stage involved screening titles and abstracts to determine eligibility. In the second stage, the full texts of the articles that successfully passed the initial stage were thoroughly examined to determine their eligibility for inclusion. Two authors conducted the study selection process separately. Disagreements regarding this process were settled through discussion.

### Data extraction

The data from the included studies were extracted and organized into Microsoft Excel 2016 spreadsheets (Microsoft Corp., Redmond, WA, USA). The extracted data included information on study identification (author, publication year), study characteristics (study locations: countries and continents, study years, sample types, cattle types, herd health status, and diagnostic techniques), and seroprevalence and molecular prevalence data (number of positive herds and number of total herds examined). The data were separately extracted by two authors. The data were thoroughly checked. Disagreements were resolved through discussion and a thorough re-examination of the original documents.

### Study quality assessment

The included articles were evaluated for potential bias using a checklist for assessing quality, which followed a previous study by Keonam *et al*. [29] and Sukon *et al*. [30]. The items evaluated were as follows: (1) Was the research objective clearly articulated? (2) Was the study’s timeframe and location clearly specified? (3) Were the samples categorized into different subgroups or explained in detail? (4) Was the sampling method thoroughly described? (5) Was the diagnostic technique and procedure clearly delineated? The item was scored using a straightforward scale system, with a value of “2” indicating affirmative, “0” indicating no, and “1” indicating doubtful. Consequently, the potential overall score for each study varied between 0 and 10.

### Statistical analysis

The herd-level seroprevalence and molecular prevalence of *C. burnetii* were separately analyzed. A herd was defined as *C. burnetii*-positive when either at least one cattle inside that herd tested positive or the pooled sample from different animals of the same herd (such as bulk tank milk) tested positive. The retrieved raw data were further arranged to simplify the statistical analysis. The data inputted into the spreadsheet were transferred to Comprehensive Meta-Analysis version 4 (Biostat, Englewood, NJ, USA) for meta-analysis. A random-effects model was used for all meta-analyses. p < 0.05 was considered statistically significant for all analyses, unless otherwise specified.

#### Overall meta-analysis

Logit transformation was used to stabilize variance before pooling data from individual studies. This transformation is defined as logit(p) = ln(p/[1−p]), where p represents the proportion, and ln denotes the natural logarithm [30]. The point estimate and its 95% confidence interval (CI) of the pooled herd-level seroprevalence and the pooled herd-level molecular prevalence of *C. burnetii* in cattle were presented following back transformation to facilitate comprehension. The heterogeneity or variance in the pooled prevalence among the studies examined was evaluated using Cochran’s Q test and the I^2^ statistic. I^2^ values of 25%, 50%, and 75% indicate low, moderate, and high degrees of heterogeneity, respectively [31]. The prediction interval (PI) was also calculated to evaluate the distribution of the true pooled prevalence in the cattle herd population. An individual study was used as the unit of analysis to calculate the overall pooled prevalence.

#### Subgroup meta-analysis

A subgroup meta-analysis was used to explore some categorical sources (factors) that may be associated with heterogeneity in the pooled herd-level seroprevalence and molecular prevalence of *C. burnetii* in cattle. These factors included the continent, sample type (bulk tank milk, serum), cattle type (beef, dairy cattle), and herd health status (healthy herd, others). Healthy herds were defined as those with healthy animals. In contrast, others were defined as those with aborted animals, those with a history of aborted animals, and those with a mixture of healthy and aborted animals. For all subgroup meta-analyses, a subgroup within the study was used as the unit of analysis. Subgroups with fewer than four studies were excluded from the analysis to prevent false-positive errors.

#### Meta-regression and cumulative meta-analysis

Subgroup meta-analyses are limited to categorical factors and can be used to analyze only one factor at a time. To overcome these problems, univariate and multivariate meta-regression analyses were used to incorporate categorical and continuous variables into the model. Univariate and multivariate meta-regression analyses were also used to determine trends in herd-level seroprevalence and herd-level molecular prevalence of *C. burnetii* in cattle over the study period. If a study did not provide the year of the study, the study year was estimated by subtracting 3 years from the publication year, representing the average time difference between the publication year and the year of the study among the included studies. In addition, a cumulative meta-analysis was performed to calculate the cumulative burden of the pooled herd-level seroprevalence and molecular prevalence of *C. burnetii* in cattle over the publication years.

#### Sensitivity analysis

A sensitivity analysis was conducted separately to assess the robustness of the pooled herd-level seroprevalence and molecular prevalence of *C. burnetii* in cattle. The results of model selection (fixed effect versus random effect model) and the unit of analysis (studies versus subgroups) were compared. A leave-one-out meta-analysis was conducted to evaluate whether each study had an excessively disproportionate impact on pooled seroprevalence and molecular prevalence. When the individual study had the greatest impact on the pooled prevalence (the study causing the highest or lowest in the pooled prevalence after removing it from the analysis), it was further used to determine its influence on the regression coefficient.

#### Publication bias

The publication bias of the pooled herd-level seroprevalence and molecular prevalence of *C. burnetii* in cattle was determined by visual assessment using funnel plots and by formal assessment using Begg’s test and Egger’s test [32, 33]. p < 0.1 suggest the existence of publication bias. The trim-and-fill method [34] was used in asymmetrical funnel plots to impute any potentially missing studies and calculate the adjusted prevalence.

## Results

### Characteristics of the included studies

**Figure-1:**
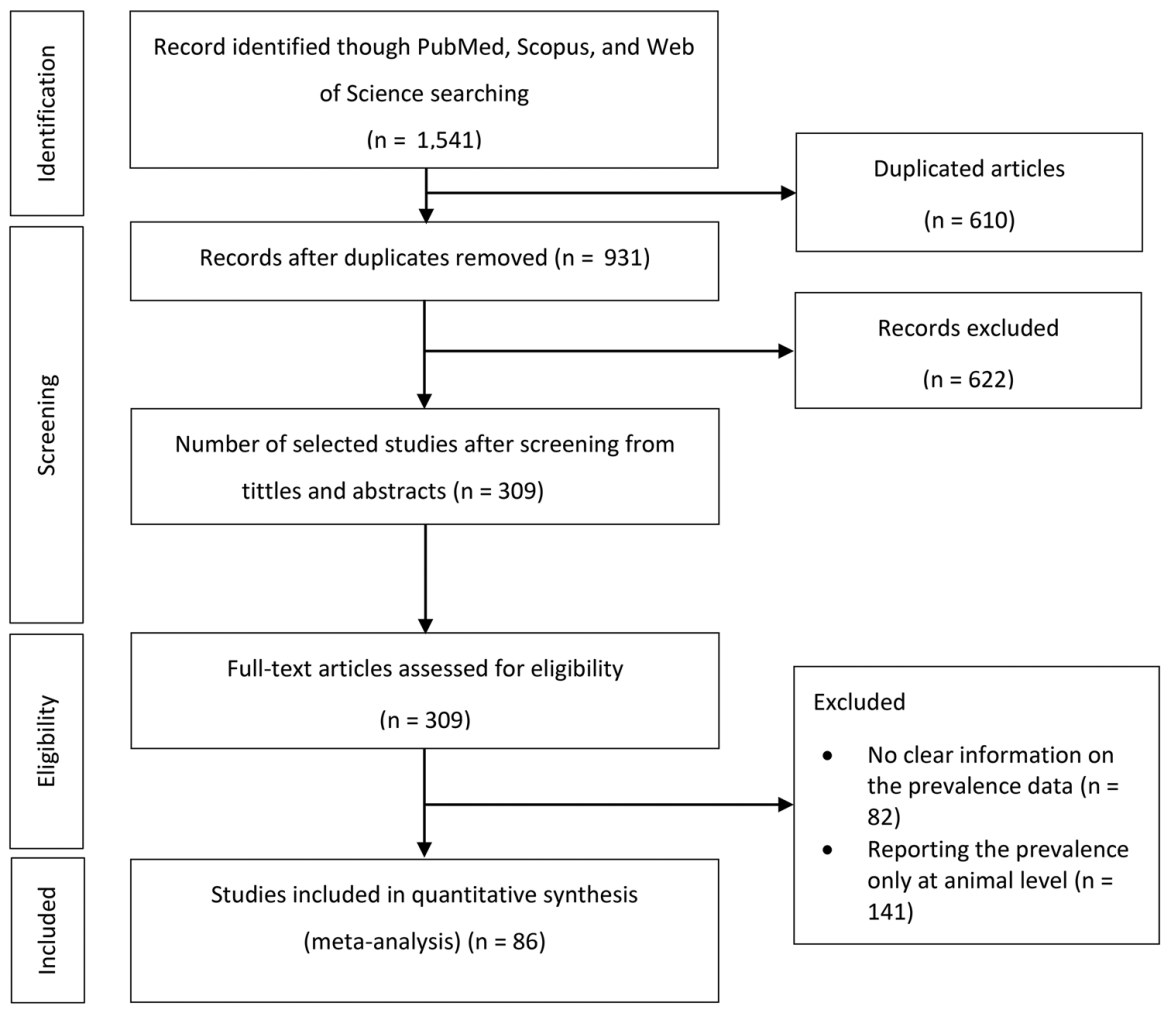
PRISMA flow chart for the study selection.

Of the 1541 citations, 86 studies with a total sample size of 38,057 cattle herds from 42 countries on six continents were included in the meta-analysis (Figure-1). The median sample size from all 86 included studies was 120 cattle herds (ranging from 4 to 3289 cattle herds). Of the 86 included studies, 55 reported herd-level seroprevalence, 18 reported herd-level molecular prevalence, and 13 reported both prevalences. Regarding studies reporting seroprevalence, the year of publication spanned from 1964 to 2023 (the study year spanned from 1961 to 2020), with a median sample size of 144 cattle herds (ranging from 4 to 3289 cattle herds). Among the 31,981 herds examined for seroprevalence, 11,045 were positive for seroprevalence. The seroprevalence data were reported for 42 countries on six continents (40 studies from Europe, nine from Africa, eight from Asia, seven from North America, three from South America, and one from Australia).

**Figure-2:**
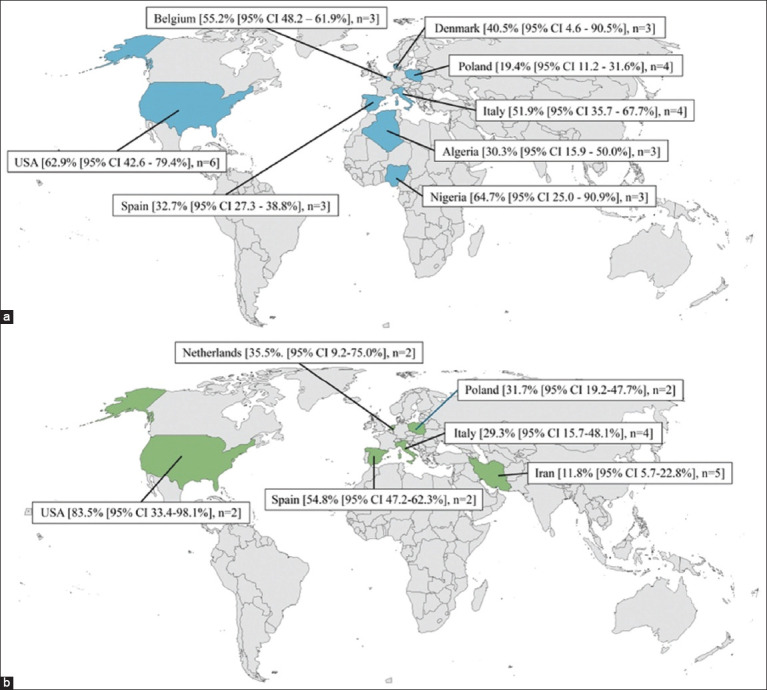
The herd-level prevalence of *Coxiella burnetii* in cattle in the countries with the most reports (a) for the seroprevalence and (b) for the molecular prevalence [Source: The base map was adopted from https://upload.wikimedia.org/wikipedia/commons/a/ac/BlankMap-World-162E.svg]

Regarding studies reporting the molecular prevalence, the year of publication spanned from 2005 to 2022 (the study year spanned from 2001 to 2021), with a median sample size of 102 cattle herds (ranging from 6 to 1077 cattle herds). Of the 6076 herds examined for molecular prevalence, 2257 were positive. The molecular prevalence data were reported from 22 countries on four continents (21 studies from Europe, six from Asia, three from North America, and one from Africa). The seroprevalence and molecular prevalence in the countries with the most reports are presented in Figure-2a and b, respectively. The included studies are listed in Table-1 [21–27, 35–113].

**Table-1:** Characteristics of the included studies.

Diagnostic techniques	Countries	Sample types	Events	Sample size	Prevalence (%)	Reference
PCR	Czech Republic	BTM	10	24	41.7	[21]
PCR	Algeria	BTM	18	200	9.0	[22]
Serology	Algeria	BTM	74	200	37.0	
PCR	Poland	BTM	33	133	24.8	[23]
Serology	Poland	Serum	89	801	11.1	
Serology	Poland	BTM	85	133	63.9	
PCR	Canada	BTM	16	74	21.6	[24]
PCR	Canada	Feces	0	31	0.0	
Serology	Canada	BTM	32	74	43.2	
PCR	Greece	BTM	156	462	33.8	[25]
Serology	Italy	Serum	63	92	68.5	[26]
PCR	Portugal	BTM	9	45	20.0	[27]
Serology	Portugal	BTM	17	45	37.8	
PCR	Iran	BTM	6	92	6.5	[35]
Serology	Spain	Serum	33	110	30.0	[36]
PCR	Spain	BTM	92	178	51.7	[37]
Serology	Spain	BTM	56	178	31.5	
Serology	India	Serum	33	35	94.3	[38]
PCR	USA	BTM	193	316	61.1	[39]
Serology	Latvia	Serum	135	1010	13.4	[40]
PCR	Latvia	BTM	27	252	10.7	
Serology	Latvia	BTM	33	252	13.1	
Serology	Germany	Serum	435	603	72.1	[41]
Serology	Nigeria	Serum	8	16	50.0	[42]
Serology	Nigeria	Serum	9	27	33.3	[43]
PCR	Turkey	BTM	5	50	10.0	[44]
Serology	Italy	Serum	36	53	67.9	[45]
Serology	Ecuador	Serum	181	386	46.9	[46]
Serology	Turkey	Serum	17	48	35.4	[47]
Serology	Thailand	Serum	8	188	4.3	[48]
PCR	Belgium	BTM	15	50	30.0	[49]
Serology	Belgium	BTM	119	206	57.8	
Serology	Lebanon	Serum	53	173	30.6	[50]
Serology	Ethiopia	Serum	7	25	28.0	[51]
Serology	Algeria	Serum	17	124	13.7	[52]
Serology	Hungary	Serum	44	44	100.0	[53]
PCR	Czech Republic	BTM	60	138	43.5	[54]
PCR	Hungary	BTM	47	126	37.3	
PCR	Serbia	BTM	8	24	33.3	
PCR	Slovakia	BTM	33	53	62.3	
PCR	Slovenia	BTM	8	16	50.0	
Serology	Czech Republic	BTM	135	138	97.8	
Serology	Hungary	BTM	123	126	97.6	
Serology	Serbia	BTM	17	24	70.8	
Serology	Slovakia	BTM	46	53	86.8	
Serology	Slovenia	BTM	10	16	62.5	
Serology	Greece	BTM	23	80	28.8	[55]
Serology	Ecuador	Serum	4	4	100.0	[56]
Serology	China	Serum	381	1140	33.4	[57]
PCR	Iran	BTM	6	6	100.0	[58]
Serology	Italy	BTM	140	402	34.8	[59]
Serology	USA	BTM	182	293	62.1	[60]
Serology	Italy	Serum	77	198	38.9	[61]
Serology	USA	BTM	266	268	99.3	[62]
PCR	France	BTM	31	37	83.8	[63]
PCR	Italy	BTM	6	12	50.0	[64]
Serology	Egypt	Serum	9	9	100.0	[65]
PCR	Hungary	BTM	10	15	66.7	[66]
Serology	Hungary	Serum	15	15	100.0	
Serology	Switzerland	Serum	270	1605	16.8	[67]
Serology	Australia	Serum	6	49	12.2	[68]
Serology	Poland	Serum	179	443	40.4	[69]
Serology	Norway	BTM	0	3749	0.0	[70]
Serology	Norway	Serum	0	55	0.0	
Serology	Iran	Serum	2	12	16.7	[71]
Serology	Iran	BTM	20	44	45.5	[72]
PCR	USA	BTM	298	316	94.3	[73]
Serology	USA	BTM	1238	4252	29.1	[74]
Serology	Northern Ireland	Serum	132	273	48.4	[75]
Serology	USA	BTM	9	24	37.5	[76]
Serology	Algeria	Serum	41	90	45.6	[77]
Serology	USA	BTM	584	1511	38.6	[78]
PCR	Netherlands	BTM	193	341	56.6	[79]
Serology	Netherlands	BTM	268	341	78.6	
Serology	Belgium	Serum	19	45	42.2	[80]
Serology	Estonia	BTM	88	324	27.2	[81]
Serology	Estonia	Serum	12	180	6.7	
PCR	Iran	BTM	14	163	8.6	[82]
Serology	Sweden	BTM	126	1537	8.2	[83]
Serology	France	BTM	2029	2948	68.8	[84]
Serology	Jordan	BTM	56	78	71.8	[85]
PCR	Sweden	BTM	250	461	54.2	[86]
Serology	Great Britian	BTM	79	373	21.2	[87]
PCR	Italy	Serum	11	102	10.8	[88]
Serology	Denmark	Serum	44	800	5.5	[89]
PCR	Italy	BTM	35	130	26.9	[90]
Serology	Portugal	BTM	55	90	61.1	[91]
PCR	Spain	BTM	56	94	59.6	[92]
PCR	Iran	BTM	6	90	6.7	[93]
PCR	Iran	BTM	5	28	17.9	[94]
Serology	Nigeria	Serum	27	28	96.4	[95]
Serology	Bangladesh	BTM	34	218	15.6	[96]
Serology	El Salvador	Serum	27	33	81.8	[97]
Serology	Spain	Serum	18	42	42.9	[98]
Serology	Republic of Ireland	BTM	110	290	37.9	[99]
Serology	Republic of Ireland	Serum	23	332	6.9	
Serology	Belgium	BTM	119	206	57.8	[100]
Serology	Chad	Serum	12	19	63.2	[101]
PCR	South Korea	BTM	108	607	17.8	[102]
Serology	Bosnia and Herzegovina	Serum	39	199	19.6	[103]
PCR	Poland	BTM	40	101	39.6	[104]
Serology	Poland	Serum	237	969	24.5	
Serology	Poland	Serum	20	358	5.6	[105]
Serology	Denmark	BTM	59	100	59.0	[106]
Serology	France	BTM	50	55	90.9	[107]
PCR	Netherlands	BTM	58	309	18.8	[108]
Serology	Netherlands	BTM	252	309	81.6	
PCR	Great Britian	BTM	57	220	25.9	[109]
Serology	Great Britian	BTM	157	221	71.0	
PCR	Italy	BTM	337	780	43.2	[110]
Serology	Greece	Serum	2	5	40.0	[111]
Serology	USA	BTM	1495	2065	72.4	[112]
Serology	Denmark	BTM	95	120	79.2	[113]

BTM=Bulk tank milk, PCR=Polymerase chain reaction, USA=United States of America

### Study quality assessment

The quality assessment tool used in this study was based on a comprehensive 10-point grading system [29, 30]. The average ± standard deviation of the overall quality scores of all included studies was 9.25 ± 0.80. The median score between 7 and 10 was 10. The results of the study quality assessment for each item are presented in Table-2 [29, 30].

**Table-2:** Study quality assessment showing the number of included studies in each category of the simple rating scale based on a checklist of five items [29, 30].

Items	No. of studies		

	Yes	No	Unsure
Was the research objective clearly articulated?	84	2	0
Was the study’s timeframe and location clearly specified?	85	0	1
Were the samples categorized into different subgroups or explained in detail?	66	0	20
Was the sampling method thoroughly described?	75	0	11
Was the diagnostic technique and procedure clearly delineated?	67	0	19

### Overall and subgroup meta-analyses

#### Herd-level seroprevalence

**Figure-3:**
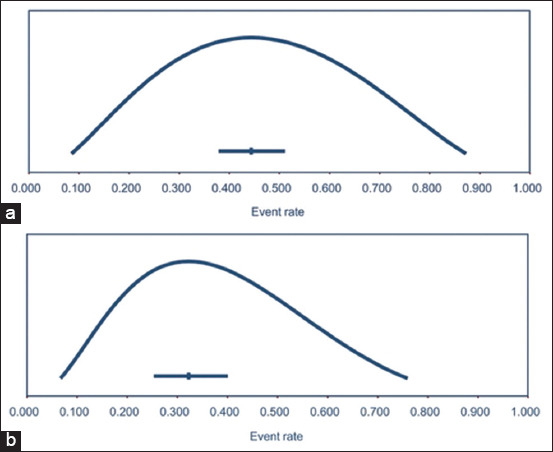
Distribution and point estimate of the herd-level prevalence of *Coxiella burnetii* in the cattle herd population (a) for the seroprevalence and (b) for the molecular prevalence.

The overall herd-level seroprevalence of *C. burnetii* in cattle was estimated to be 44.4% (95% CI: 37.9%–51.1%; PI: 8.5%–87.2%), with high heterogeneity among the included studies (Cochran’s Q = 5382.1; p < 0.001; I^2^ = 98.8%; n = 68 studies) (Figure-3a). For the subgroup meta-analysis of four sources (continents, sample types, cattle types, and herd health status), only sample types and cattle types showed statistically significant differences among subgroups. For sample types, herd-level seroprevalence was significantly higher in bulk tank milk (BTM) than in serum (52.3% [95% CI: 43.8%–60.7%] vs. 32.7% [95% CI: 26.1%–40.1%], respectively). For cattle types, herd-level seroprevalence was significantly higher in dairy cattle than in beef cattle (49.0% [95% CI: 41.9%–56.2%] vs. 14.5% [95% CI: 5.8%–32.1%], respectively). The herd-level seroprevalence ranged from 33.4% (95% CI: 21.9%–48.6%) in Asia to 55.1% (95% CI: 38.2%–70.8%) in North America. Regarding cattle herd health status, herd-level seroprevalence in healthy herds was not significantly different from that in herds associated with abortion (43.9% [95% CI: 37.6%–50.3%] vs. 32.4% [95% CI: 19.0%–49.6%], respectively). The results of the overall and subgroup meta-analyses of the herd-level seroprevalence of *C. burnetii* in cattle are presented in Table-3.

**Table-3:** Overall and subgroup meta-analyses of herd-level seroprevalence of *Coxiella burnetii* in cattle.

Categories	No. of studies or subgroups	Prevalence (%)			Heterogeneity			p-value for subgroup differences
	
		Estimate	95% CI	PI	Q	p-value	I^2^ (%)	
Overall	68	44.4	37.9–51.1	8.5–87.2	5382.1	<0.001	98.8	
Continents^a^								0.268
Africa	9	44.9	30.7–60.0	9.9–85.7	54.9	<0.001	85.4	
Asia	9	33.4	21.9–48.6	5.2–82.0	137.4	<0.001	94.2	
Europe	56	39.9	31.8–48.6	4.7–90.0	4091.0	<0.001	98.7	
North America	8	55.1	38.2–70.8	9.4–93.5	1180.3	<0.001	99.4	
Sample types								0.001
BTM	40	52.3	43.8–60.7	11.3–90.4	3313.5	<0.001	98.8	
Serum	46	32.7	26.1–40.1	5.9–79.1	1545.9	<0.001	97.1	
Cattle types^b^								0.001
Beef	10	14.5	5.8–32.1	0.4–88.8	294.5	<0.001	96.9	
Dairy	63	49.0	41.9–56.2	9.7–89.6	4962.4	<0.001	98.8	
Herd health status								0.211
Healthy	74	43.9	37.6–50.3	8.5–86.8	4862.8	<0.001	98.5	
Others^c^	12	32.4	19.0–49.6	2.7–89.2	457.9	<0.001	97.6	

a Two continents were excluded from the analysis due to the low number of studies or subgroups (Australia, n = 1; South America, n = 3).

b Studies or subgroups of unspecified cattle types (n = 13) were excluded from the analysis.

c Others included herds with aborted animals, those with a history of aborted animals or mixing of healthy and aborted animals in the same herd. BTM=Bulk tank milk, CI=Confidence interval, PI=Prediction interval

#### Herd-level molecular prevalence

The overall herd-level molecular prevalence of *C. burnetii* in cattle herds was estimated to be 32.3% (95% CI: 25.3%–40.01%; PI: 6.7%–76.0%), with high heterogeneity among the included studies (Cochran’s Q = 886.9; p < 0.001; I^2^ = 96.6%; n = 31 studies) (Figure-3b). Only two characteristics (continents and herd health status) were included in the subgroup analysis. The herd-level molecular prevalence differed significantly between continents (p < 0.001). The herd-level molecular prevalence ranged from 12.8% (95% CI: 7.1%–21.9%) in Asia to 37.8% (95% CI: 32.6%–44.4%) in Europe and 70.0% (95% CI: 36.3%–90.5%) in North America. Regarding cattle herd health status, the herd-level molecular prevalence in healthy herds was not significantly different from that in herds associated with abortion (40.1% [95% CI: 32.5%–48.3%] vs. 27.5% [95% CI: 14.6%–45.8%], respectively). The results of the overall and subgroup meta-analyses of the herd-level molecular prevalence of *C. burnetii* in cattle are presented in Table-4.

**Table-4:** Overall and subgroup meta-analyses of the herd-level molecular prevalence of *Coxiella burnetii* in cattle.

Categoriesa	No. of studies or subgroups	Prevalence (%)			Heterogeneity			p-value for subgroup differences
	
		Estimate	95% CI	PI	Q	p-value	I^2^ (%)	
Overall	31	32.3	25.3–40.01	6.7–76.0	886.9	<0.001	96.6	
Continents^b^								<0.001
Asia	6	12.8	7.1–21.9	1.8–54.2	26.9	<0.001	81.4	
Europe	26	37.8	31.6–44.4	13.3–70.6	363.8	<0.001	93.1	
North America	6	70.0	36.3–90.5	1.5–99.7	139.9	<0.001	96.4	
Herd health status								0.197
Healthy	31	40.1	32.5–48.3	9.9–80.4	717.9	<0.001	95.8	
Others^c^	8	27.5	14.6–45.8	2.2–86.3	126.3	<0.001	94.5	

^a^The sample and cattle types did not undergo subgroup analysis. For sample types, of 39 studies or subgroups, almost all studies or subgroups (n = 37) were from BTM, and only one study or subgroup was from serum and feces each. For cattle types, out of 39 studies or subgroups, 37 studies or subgroups were from dairy cattle, and only two studies or subgroups were from unspecified cattle types.

b Three continents were excluded from the analysis due to the lack of studies or subgroups (Australia, n = 0; Africa, n = 1; South America, n=0).

c Others included herds with aborted animals, those with a history of aborted animals or mixing of healthy and aborted animals in the same herd. CI=Confidence interval, PI=Prediction interval

### Meta-regression and cumulative meta-analysis

#### Herd-level seroprevalence

The results of the meta-regression analysis showed that the trend of the herd-level seroprevalence of *C. burnetii* in cattle did not change significantly over time (regression coefficient was −0.01 [95% CI: −0.03–0.01, p = 0.330] according to the univariate analysis and 0.01 [95% CI: −0.02–0.04, p = 0.669] according to the multivariate analysis). Table-5 presents the regression coefficient and associated odds ratio (OR) of each factor from the univariate and multivariate meta-regression analyses. The univariate meta-regression revealed that two factors (sample type and cattle type) were significantly different (p = 0.001 for sample type and p < 0.001 for cattle type) between subgroups. However, after accounting for other factors in the multivariate meta-regression, only one factor (cattle type) was significantly different (p = 0.004). Namely, the adjusted OR of the herd-level seroprevalence in dairy cattle was 4.03 (95% CI: 1.58–10.29) times higher than that in beef cattle.

**Table-5:** Univariate and multivariate meta-regression analyses of herd-level seroprevalence of *Coxiella burnetii* in cattle.

Categories	Univariate meta-regression			Multivariate meta-regression		
	
	Coefficient (95% CI)	Crude OR (95% CI)	p-value	Coefficient (95% CI)	Adjusted OR (95% CI)	p-value
Study year	−0.01 (−0.03–0.01)	0.99 (0.97–1.01)	0.330	0.01 (−0.02–0.04)	1.01 (0.98–1.04)	0.669
Continents						
Africa	0.62 (−0.55–1.79)	1.86 (0.58–5.98)	0.299	0.65 (−0.69–1.98)	1.91 (0.50–7.26)	0.341
Europe	0.27 (−0.60–1.14)	1.31 (0.55–3.13)	0.545	0.06 (−0.87–1.00)	1.06 (0.42–2.71)	0.895
North America	0.93 (−0.23–2.09)	2.53 (0.79–8.07)	0.117	0.44 (−1.35–2.22)	1.55 (0.26–9.23)	0.638
Asia	Reference			Reference		
Sample types						
BTM	0.81 (0.34–1.28)	2.25 (1.40–3.59)	0.001	0.49 (−0.20–1.19)	1.64 (0.82–3.27)	0.165
Serum	Reference			Reference		
Cattle types						
Dairy	1.67 (0.88–2.46)	5.34 (2.42–11.76)	< 0.001	1.39 (0.46–2.33)	4.03 (1.58–10.29)	0.004
Beef	Reference			Reference		
Herd health status						
Healthy	0.51 (−0.18–1.19)	1.66 (0.83–3.30)	0.149	0.54 (−0.32–1.14)	1.71 (0.72–4.06)	0.221
Others	Reference			Reference		

CI=Confidence interval, OR=Odds ratio

**Figure-4:**
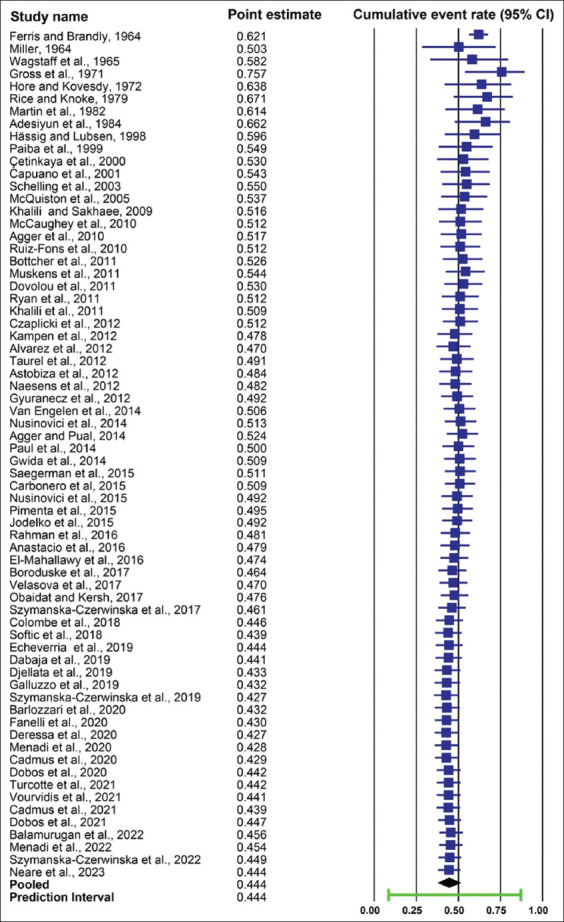
Cumulative evidence of pooled herd-level seroprevalence of *Coxiella burnetii* in cattle.

The cumulative evidence of pooled herd-level seroprevalence of *C. burnetii* in cattle gradually decreased from 62.1% in 1964 to 50.9% in 2011. From 2012 to 2023, the cumulative evidence of pooled seroprevalence fluctuated between 42.7% and 52.0% (Figure-4).

#### Herd-level molecular prevalence

Although the trend of the herd-level molecular prevalence of *C. burnetii* in cattle decreased significantly over time according to the univariate analysis (the regression coefficient was −0.11 [95% CI: −0.16–−0.05, p < 0.001]), after accounting for other factors in multivariate analysis, the trend of the molecular prevalence was inconclusive (the regression coefficient was −0.05 [95% CI: −0.11–0.00, p = 0.049]). Table-6 shows the additional regression coefficient and its associated OR for each factor from the univariate and multivariate meta-regression analyses. Univariate and multivariate analyses revealed that only one factor (continents) exhibited statistical significance. After accounting for other factors, the adjusted ORs of the herd-level molecular prevalence were 4.01 (95% CI: 1.89–8.47) in Europe and 12.43 (95% CI: 4.37–35.35) in North America compared with Asia.

**Table-6:** Univariate and multivariate meta-regression analyses of herd-level molecular prevalence of *Coxiella burnetii* in cattle.

Categories	Univariate meta-regression			Multivariate meta-regression		
	
	Coefficient (95% CI)	Crude OR (95% CI)	p-value	Coefficient (95% CI)	Adjusted OR (95% CI)	p-value
Study year	−0.11 (−0.16–−0.05)	0.90 (0.85–0.95)	<0.001	−0.05 (−0.11–0.00)	0.95 (0.90–1.00)	0.049
Continents						
Europe	1.41 (0.64–2.18)	4.08 (1.89–8.81)	<0.001	1.39 (0.64–2.14)	4.01 (1.89–8.47)	<0.001
North America	2.97 (1.97–3.96)	19.39 (7.15–52.67)	<0.001	2.52 (1.47–3.57)	12.43 (4.37–35.37)	<0.001
Asia	Reference			Reference		
Herd health status						
Healthy	0.60 (−0.15–1.36)	1.83 (0.86–3.90)	0.117	0.31 (−0.33–0.96)	1.37 (0.72–2.62)	0.341
Others	Reference			Reference		

CI=Confidence interval, OR=Odds ratio

**Figure-5:**
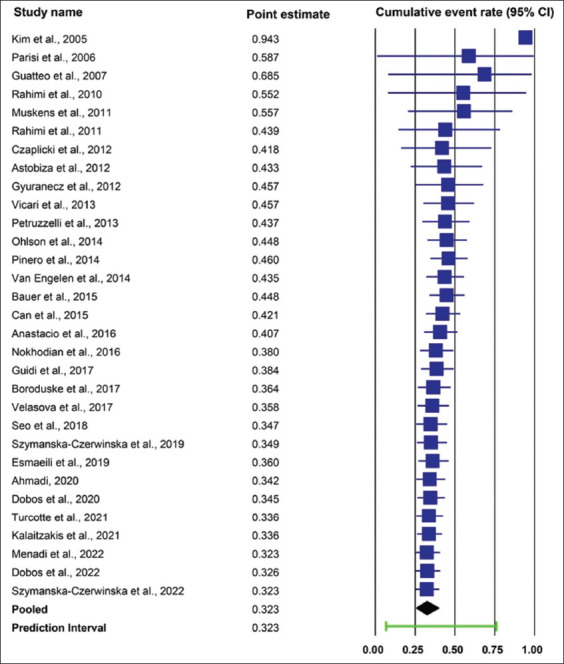
Cumulative evidence of pooled herd-level molecular prevalence of *Coxiella burnetii* in cattle.

The cumulative evidence of the pooled herd-level molecular prevalence of *C. burnetii* in cattle varied between 42.1% and 94.3% from 2005 to 2015. However, from 2017 to 2022, the cumulative evidence of pooled herd-level molecular prevalence fluctuated slightly between 32.3% and 38.4% (Figure-5).

### Sensitivity analysis

The sensitivity analysis results for herd-level seroprevalence and molecular prevalence of *C. burnetii* in cattle are presented in Table-7 [35, 62, 70, 73]. The pooled estimates of both seroprevalence and molecular prevalences were robust to model selection, the unit of analysis, and the influence of an individual study. However, the regression coefficient of the molecular prevalence was sensitive in the study by Kim *et al*. [73]. After removing this study from the univariate meta-regression analysis, the regression coefficient changed from a statistically significant difference (−0.11 [95% CI: −0.16–−0.05, p < 0.001]) to a non-significant difference (−0.02 [95% CI: −0.08–0.03, p = 0.416]).

**Table-7:** Sensitivity analysis to assess the robustness of herd-level seroprevalence and molecular prevalence estimates of *Coxiella burnetii* in cattle.

Categories	No. of studies or subgroups	Prevalence (%)	

		Estimate	95% CI
Seroprevalence			
Model			
Fixed effects	68	40.4	39.8–41.1
Random effects	68	44.4	37.9–51.1
Units of analysis			
Studies	68	44.4	37.9–51.1
Subgroups	86	42.1	36.3–48.2
Leave-one-out analysis			
The lowest prevalence^a^	67	42.9	36.5–49.6
The highest prevalence^b^	67	45.7	39.1–52.4
Molecular prevalence			
Model			
Fixed effects	31	38.2	36.8–39.6
Random effects	31	32.3	25.3–40.1
Units of analysis			
Studies	31	32.3	25.3–40.1
Subgroups	39	37.3	30.5–44.7
Leave-one-out analysis			
The lowest prevalence^c^	30	29.6	23.5–36.4
The highest prevalence^d^	30	33.6	26.4–41.6

a Removed the study of Gross *et al*. [62].

b Removed the study of Kampen *et al*. [70].

c Removed the study of Kim *et al*. [73]. dRemoved the study of Ahmadi *et al*. [35]. CI=Confidence interval

### Publication bias

For the analysis of the herd-level seroprevalence of *C. burnetii* in cattle, Begg’s test (p = 0.117) and Egger’s test (p = 0.899) indicated no publication bias. However, five studies were missing from Duval and Tweedie’s trim-and-fill method. After these missing studies were imputed, the estimated herd-level seroprevalence changed somewhat from 44.4% (95% CI: 37.8%–51.1%) to 40.4% (95% CI: 34.2%–46.9%). Regarding the herd-level molecular prevalence of *C. burnetii* in cattle, Begg’s test (p = 0.786) and Egger’s test (p = 0.196) indicated no publication bias. Duval and Tweedie’s trim and fill method revealed no studies with missing data.

## Discussion

Our study aimed to estimate the global herd-level prevalence of *C. burnetii* in cattle, explore the sources of heterogeneity associated with the pooled prevalence, and determine the trend of the pooled prevalence over time. We estimated the global prevalence of *C. burnetii* in 38057 cattle herds from 86 included studies from 42 countries. Seroprevalence and molecular prevalence differ inherently. Seroprevalence tests, such as enzyme-linked immunosorbent assay (ELISA) and complement fixation test are used to detect antibody titers. A positive seroprevalence test indicates previous exposure to or infection by an infectious agent. In contrast, PCR is commonly used to detect the antigens of infectious agents. A positive molecular prevalence test indicates current infections. Therefore, we separately analyzed herd-level seroprevalence and herd-level molecular prevalence of *C. burnetii* in cattle.

In our study, the global herd-level seroprevalence and molecular prevalence of *C. burnetii* in cattle were estimated to be 44% and 32%, respectively. Thus, nearly half of the cattle herds were exposed to *C. burnetii*, and approximately one-third had active *C. burnetii* infections. High herd-level seroprevalence and high herd-level molecular prevalence indicate a risk to public health worldwide. Management of *C. burnetii* infection in cattle is critical for successful disease control. Management programs should include the diagnosis of *C. burnetii* in individual animals and infected herds and the surveillance and control of Q fever in cattle farms [11].

We found high heterogeneity in the estimates of pooled herd-level seroprevalence and molecular prevalence of *C. burnetii* in cattle, as indicated by Q tests and I^2^ statistics. Thus, the pooled herd-level seroprevalence and molecular prevalence in the cattle herd population may vary substantially (not resulting from chance alone). High heterogeneity can also be realized from the PI. This interval indicates the extent to which the pooled prevalence in the population varies [114, 115]. In our study, the PIs of the pooled herd-level seroprevalence and the pooled herd-level molecular prevalence were approximately 9%–87% and 7%–76%, respectively. The high heterogeneity of the meta-analysis from prevalence studies (also known as proportional meta-analysis) is a common phenomenon due to the differences in the nature of the included studies [116]. The individually included studies are prevalence studies and are usually conducted in different contexts, such as in different cattle populations, study periods, and geographical regions. These differences can cause high heterogeneity in the pooled estimate. Although individual prevalence studies have been conducted in different contexts, combining the evidence from these studies is particularly useful for determining the global disease burden and trend of disease over time [117].

We explored sources of heterogeneity in the pooled herd-level seroprevalence and molecular prevalence of *C. burnetii* in cattle using subgroup meta-analysis and univariate and multivariate meta-regression analyses. The results of the subgroup analysis and univariate meta-regression analysis are interpreted similarly because both methods analyze only one source or factor at a time. However, the results from the subgroup analysis are presented as the percentage prevalence, whereas those from the univariate meta-regression are presented as crude ORs (crude ORs). Five sources (factors) of heterogeneity (study years, continents, sample types, cattle types, and herd health status) were analyzed for seroprevalence, but only three sources (study years, continents, and herd health status) were analyzed for molecular prevalence due to data unavailability.

Regarding the continents of the included studies, the herd-level seroprevalence of *C. burnetii* in cattle did not significantly differ among the continents (33% in Asia, 40% in Europe, 45% in Africa, and 55% in North America). Our data show that seroprevalence was high on all four continents. This result indicates that Q fever is abundant in cattle herds worldwide. The heterogeneity of pooled seroprevalence was still high for each continent. This finding may reflect differences in herd-level seroprevalence between countries within each continent or between studies within the same country. For example, in Asia, herd-level seroprevalence was as high as 94% in India [38], compared with 4% in Thailand [48]. In the USA, herd-level seroprevalence was as high as 99% [62] and 29% in another study by Martin *et al*. [74]. Discrepancies in herd-level seroprevalence and molecular prevalence of *C. burnetii* among continents were observed. Namely, the molecular prevalence significantly differed across continents (13% in Asia, 38% in Europe, and 70% in North America). The molecular prevalence of this disease was low in Asia and high in North America. However, the included studies or subgroups were small (n = 6 each) for both continents. Additional data from these continents are required to provide robust evidence. Differences in herd-level molecular prevalence among countries (or even within the same country) on the same continent were also observed. For example, in Iran, the herd-level molecular prevalence was as low as 7% in one study [93] and as high as 100% in another study [58].

Regarding sample types, the herd-level seroprevalence from BTM samples was significantly higher than that from serum samples (53% vs. 33% or crude OR = 2.25, [95% CI: 1.40–3.59], p = 0.001). However, in the multivariate meta-regression model, the seroprevalence between BTM and serum was not significantly different (adjusted OR = 1.64, [95% CI: 0.82–3.27], p = 0.165). The differences in the statistical results between the univariate and multivariate meta-regression analyses can be explained as follows. In the univariate analysis, we considered only one factor (sample type) and ignored other factors that could influence the pooled estimate. However, in the multivariate analysis, when analyzing the sample types, we accounted or adjusted for other factors (study years, continents, cattle types, and herd health status). We also obtained the adjusted OR from the analysis model. Therefore, the results of the multivariate meta-regression analysis are more applicable in real-world situations than univariate meta-regression analysis. We also found that almost all included studies used ELISA to determine the seroprevalence of BTM and serum. Determining BTM antibody concentrations using ELISA is useful for predicting within-herd seroprevalence and large-scale screening programs to detect previously infected herds [11, 107]. Subgroup meta-analysis and meta-regression analysis of sample types were not performed due to data limitations. However, the molecular prevalence of BTM in our study was 39.1% (95% CI: 32.0%–46.6%; n = 37 studies or subgroups). This is similar to a recent meta-analysis on bovine dairy herds [118]. Rabaza *et al*. [118] reported that the molecular prevalence of *C. burnetii* from BTM in dairy cattle herds was 37.0% (95% CI: 25.2%–49.5%; n = 17 studies).

Regarding cattle type, the herd-level seroprevalence of *C. burnetii* was significantly higher in dairy cattle than beef cattle (49% vs. 19%, respectively, or approximately 30% difference) according to univariate and multivariate meta-regression analyses. Thus, after accounting for other factors (or, in other words, after adjusting for other factors that were the same for both dairy and beef cattle), the pooled herd-level seroprevalence was higher in dairy cattle than beef cattle. Our results for the seroprevalence at the herd level were similar to those at the animal level because the animal-level seroprevalence of *C. burnetii* was also higher in dairy cattle than beef cattle [89]. Therefore, dairy cattle herds are at greater risk than beef cattle herds. In addition, *C. burnetii* can be shed through milk and is a potential source of infections in humans [15, 118, 119]. Due to data limitations, the subgroup meta-analysis and meta-regression analysis of cattle types were not performed.

Regarding herd health status, we categorized herd health status into two categories: (1) Healthy (the herds with healthy animals) and others (the herds with aborted animals, with a history of aborted animals, or with a mixture of healthy and aborted animals in the same herds). We found that neither the herd-level seroprevalence nor the herd-level molecular prevalence of *C. burnetii* differed significantly between the healthy and the other herds (44% vs. 32% for the seroprevalence and 40% vs. 28% for the molecular prevalence, respectively). These results indicate that exposure to or current infection with *C. burnetii* may be common in healthy herds because most infected cattle are asymptomatic or not aborted, as described by many authors [1, 8, 11]. Therefore, surveillance and monitoring of *C. burnetii* in healthy cattle herds are necessary to control Q fever in these herds [11].

The trend of the global herd-level seroprevalence of *C. burnetii* in cattle herds did not change significantly over time (from both univariate and multivariate meta-regression analyses). These results indicate that *C. burnetii* remains a problem for cattle herds worldwide. According to the results of the cumulative meta-analysis, the cumulative evidence of herd-level seroprevalence was substantial>50% between 1964 and 2011; from 2012 to 2023, the cumulative evidence was also substantially high and fluctuated between 42% and 53%, with the most recent cumulative evidence in 2023 accounting for approximately 40%. This high cumulative seroprevalence indicates that the Q fever burden in cattle herds worldwide has persisted.

Although the trend of the herd-level molecular prevalence of *C. burnetii* in cattle decreased significantly over time according to the univariate meta-regression analysis (p < 0.001), this statistical conclusion may not be valid because it is not robust. We performed a further sensitivity analysis to assess the robustness of this statistical conclusion by testing the influence of the individual studies. After we removed one study with the greatest influence (determined by a leave-one-out meta-analysis) on the molecular prevalence [73] from the univariate meta-regression model, the trend of the molecular prevalence was not statistically significant (p = 0.416). In addition, the results of the multivariate meta-regression analysis indicated that the trend of the molecular prevalence was still inconclusive because P value from the statistical test fell at the borderline between the accepting and rejecting regions of the null hypothesis (p = 0.049) after accounting for continents and the herd health status in the model. The trend of herd-level molecular prevalence of *C. burnetii* in cattle herds should be interpreted with caution. Moreover, the cumulative evidence on the molecular prevalence was relatively stable (little fluctuation) from 2017 to 2022. Therefore, additional information is required to clarify the trend of herd-level molecular prevalence.

Regarding publication bias, our results from Begg’s test and Egger’s test indicated that publication bias was absent for either herd-level seroprevalence or herd-level molecular prevalence. Publication bias may not have interfered with our interpretation.

This study has several limitations. First, we included only studies with full text published in English and indexed in PubMed, Scopus, and Web of Science. Therefore, we excluded studies published in other languages or those not indexed in these three databases. Regarding the study locations, the number of included studies is limited on some continents. For example, few studies have reported herd-level seroprevalence in Australia (n = 1) or South America (n = 3). Only one study reported herd-level molecular prevalence in Africa, and no study reported herd-level molecular prevalence in Australia or South America. These limitations are not feasible for the subgroup meta-analysis of these continents. The global prevalence of *C. burnetii* in cattle herds should be interpreted with caution. Additional data from further studies in other regions may alter the pooled herd-level seroprevalence and molecular prevalence of *C. burnetii* in cattle herds. As the objective of our study was limited to herd-level prevalence, a substantial number of studies that reported solely animal-level prevalence data were excluded from the study. Therefore, a systematic review and meta-analysis of the prevalence of *C. burnetii* in cattle at the animal level is required.

## Conclusion

This study used a random-effects meta-analysis to estimate the global herd-level seroprevalence and molecular prevalence of *C. burnetii* in cattle. The global herd-level seroprevalence was estimated at 44%, with high heterogeneity. The distribution of true pooled herd-level seroprevalence in the cattle herd population varied greatly (9%–87%). The global herd-level molecular prevalence was estimated at 32%, with high heterogeneity. The distribution of the true pooled herd-level molecular prevalence in the cattle herd population also varied greatly (possibly ranging from 7% to 76%). The trend of the seroprevalence of *C. burnetii* in cattle herds did not change significantly over time. Based on the available data, the interpretation of the trend in herd-level molecular prevalence of *C. burnetii* in cattle herds was not valid due to the significant influence of a particular study. The high herd-level seroprevalence and molecular prevalence of *C. burnetii* in cattle indicate potential risks to human health.

## Authors’ Contributions

AK: Conceptualization, methodology, formal analysis, investigation, data curation, writing – original draft, writing – review and editing, and project administration. NHN: Conceptualization, writing – review and editing, and supervision. SR: Conceptualization, writing – review and editing, and supervision. CT: Conceptualization, writing – review and editing, and supervision. SS: Conceptualization, writing – review and editing, and supervision. MY: Conceptualization, writing – review and editing, and supervision. PS: Conceptualization, methodology, formal analysis, investigation, data curation, writing – original draft, writing – review and editing, and supervision. All authors have read and approved the final manuscript.
